# N-acetylated amino acids: An overlooked layer of metabolic regulation

**DOI:** 10.1016/j.molmet.2026.102425

**Published:** 2026-07-16

**Authors:** Dirk Weber, Achim Bub

**Affiliations:** 1 Institute of Sports and Sports Science, Karlsruhe Institute of Technology, Karlsruhe, Germany; 2 Department of Nutritional Epidemiology and Physiology, Max Rubner-Institute, Karlsruhe, Germany

**Keywords:** N-acetylated amino acids, Acetyl-CoA, Amino acids, Metabolic regulation

## Abstract

N-acetylated amino acids (Ac-AAs) have been repeatedly reported in metabolomics studies of high-intensity exercise, cold-exposed brown adipose tissue (BAT), and various pathological conditions. Despite their recurrent detection, the origins and physiological functions of Ac-AAs remain poorly understood, and evidence is fragmented across diverse scientific disciplines. While Ac-AAs have traditionally been attributed to the degradation of N-terminally acetylated proteins, this mechanism alone cannot fully account for their diversity and context-dependent regulation. Instead, accumulating evidence supports a model in which Ac-AA formation is driven by elevated intracellular acetyl-CoA and amino acid availability. Under these conditions, Ac-AA formation may represent a previously unrecognized metabolic mechanism involved in acetyl-CoA and amino acid homeostasis. In this review, we provide an overview of Ac-AA alterations across physiological contexts, synthesize current evidence on their origins, regulation, and physiological functions, and propose a mechanistic framework for the role of Ac-AAs in metabolic regulation. By integrating findings across diverse scientific disciplines, this review establishes a foundation for a more consistent interpretation of Ac-AAs across physiological and pathological contexts.

## 1 Introduction

Metabolomics studies have repeatedly reported alterations in N-acetylated amino acids (Ac-AAs) across diverse physiological contexts, such as high-intensity exercise or cold exposure in brown adipose tissue (BAT) [1,2]. Likewise, Ac-AAs have been observed in various pathological conditions, including cancer, metabolic diseases, chronic kidney disease (CKD), and inborn errors of metabolism [[3], [4], [5], [6]]. Acetylation reactions are central to human physiology, ranging from post-translational protein acetylation to the acetylation of small molecules involved in metabolic regulation and detoxification [7]. Accordingly, certain Ac-AAs serve well-established physiological functions, such as N-acetylaspartate (NAA), a highly abundant neuronal metabolite [8], or N-acetylglutamate (NAG), an essential allosteric cofactor for carbamoyl phosphate synthetase I in the urea cycle [9]. However, for most Ac-AAs no specific metabolic origins and physiological functions have been demonstrated. Consequently, the presence of Ac-AAs in metabolomics studies is often attributed to the degradation of N-terminally acetylated proteins, which currently represents the only established source for a broad spectrum of Ac-AAs.

Several lines of evidence indicate that protein degradation alone may not fully account for the recurrent detection of multiple Ac-AAs. Accordingly, various alternative hypotheses for the physiological roles of Ac-AAs have been proposed, each supported by distinct lines of evidence and emerging from different scientific disciplines and experimental contexts. Suggested physiological functions include buffering excess acetyl-CoA or serving as reservoirs for two-carbon units, with potential effects on protein acetylation and lipid synthesis [[10], [11], [12]]. Moreover, several studies have proposed signaling functions for individual or multiple Ac-AAs, highlighting their relevance for exerkine research [12,13]. Nevertheless, interpretations of the physiological roles of Ac-AAs remain fragmented across diverse scientific disciplines and lack integration within a unifying mechanistic framework.

In this review, we integrate evidence from physiological contexts in which Ac-AAs have been observed, including high-intensity exercise and cold exposure in BAT, and briefly summarize findings on Ac-AAs reported across pathological conditions. We then examine mechanisms of formation and degradation, discuss potential physiological functions, and integrate these observations into a coherent mechanistic framework for Ac-AAs in metabolic regulation. By integrating these findings, this review provides a conceptual foundation for interpreting Ac-AAs in metabolomics studies and for guiding future experimental investigations.

## 2 Physiological and pathological contexts of Ac-AAs

### 2.1 Ac-AAs in high-intensity exercise

**Figure 1:**
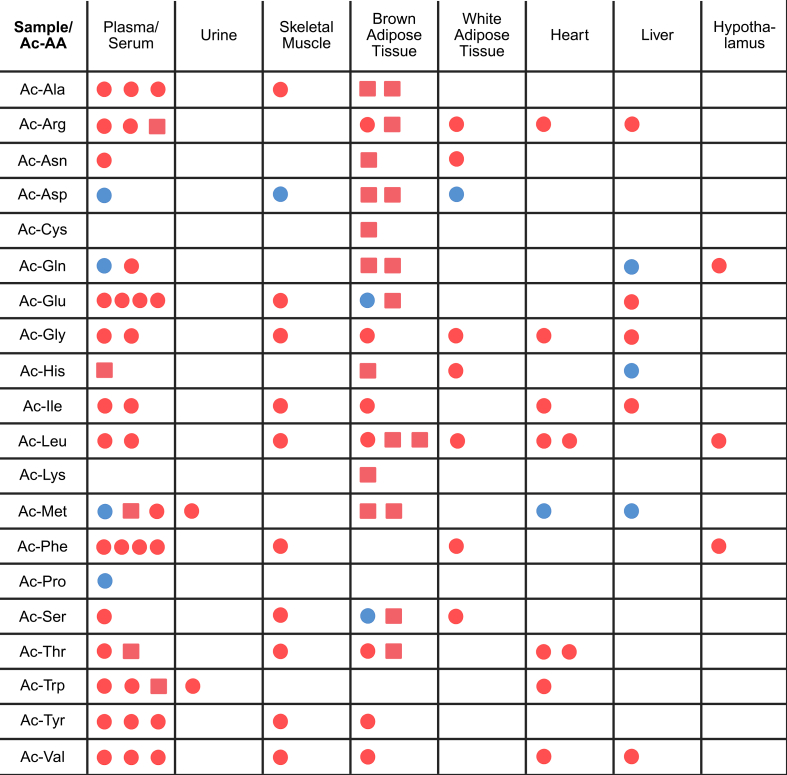
**N-acetylated amino acids across metabolomics studies of acute exercise and cold-exposed BAT.** Circles represent exercise studies, whereas squares represent mouse studies of cold-exposed brown adipose tissue (BAT). Red symbols indicate increases and blue symbols decreases in Ac-AA levels. Ac-AAs are shown using standard three-letter amino acid abbreviations. Columns indicate analyzed tissues across studies. Not all sample types were investigated in every study; for example, urine was analyzed in only one study. Several tissues, including hypothalamus, liver, white adipose tissue, and skeletal muscle, originate from the same exercise study. The figure is not intended to be comprehensive.

Metabolomics studies have repeatedly reported increases in multiple Ac-AAs following acute bouts of exercise [2,11,[14], [15], [16], [17], [18], [19], [20]]. These studies span both human and mouse models, and assess Ac-AAs across multiple biological compartments, including circulation, urine, skeletal muscle, adipose tissue, and the hypothalamus (see Figure 1 and Supplementary Table 1). Across studies, N-acetylated derivatives of nearly all proteinogenic amino acids have been reported to change in response to acute exercise, alongside frequent observations of N-acetylated non-proteinogenic amino acids such as N-acetyltaurine and N-acetylcitrulline. The vast majority of Ac-AAs show an overall tendency to increase in response to exercise. In contrast, a small subset, including N-acetylmethionine, shows inconsistent changes across studies, whereas N-acetylproline and N-acetylaspartate have only been reported to decrease. Following acute exercise, Ac-AAs return toward baseline levels during early post-exercise recovery [11,21].

Notably, Ac-AAs are most frequently reported following high exercise intensities or under conditions of substantial metabolic stress, such as ultramarathon events [15], high-intensity interval swimming [20], or treadmill running to exhaustion [19]. In line with this, we recently demonstrated that multiple Ac-AAs increased only in response to continuous vigorous exercise at 75% VO_2_peak, whereas no increase was observed at a moderate intensity of 50% VO_2_peak [11]. These findings indicate that systemic increases in Ac-AAs may only emerge at higher exercise intensities or under conditions of substantial metabolic stress. As most exercise metabolomics studies report only a limited subset of Ac-AAs, they are often overlooked within the broader spectrum of exercise-responsive metabolites. Consequently, Ac-AAs are rarely discussed or interpreted within the context of sports and exercise physiology, with few studies to date addressing their potential functional relevance [11]. Collectively, existing studies establish a clear association between high-intensity exercise and systemic increases in Ac-AAs, yet do not clarify the metabolic origin or physiological functions of these metabolites.

### 2.2 Ac-AAs in cold-exposed brown adipose tissue

Cold exposure has repeatedly been shown to alter multiple Ac-AAs in brown adipose tissue (BAT), including non-proteinogenic derivatives such as N-acetylornithine [1,10,12,22]. To date, this evidence comes primarily from mouse studies, where direct tissue sampling is feasible (see Figure 1 and Supplementary Table 1). Across studies, Ac-AAs were consistently increased in response to cold acclimation, with up to 4,000-fold higher relative abundances in BAT than in circulation [10]. Notably, Pileggi et al. reported that multiple Ac-AAs decreased during cold deacclimation, defined as the transition from cold exposure to thermoneutral conditions. In contrast, N-acetyllysine was the only Ac-AA that increased in response to cold deacclimation. The same study quantified individual Ac-AAs and reported BAT Ac-AA concentrations in the range of 0.001–1.0 μmol/g tissue (unit confirmed by personal communication with the authors) [12]. Using arteriovenous metabolomics, Park et al. demonstrated, in a small subset of Ac-AAs, that BAT can both release Ac-AAs into and take them up from the circulation [23].

N-acetylaspartate (NAA) is primarily known as a highly abundant neuronal metabolite, yet it also represents the most extensively studied Ac-AA in BAT. The synthesis of NAA is catalyzed by aspartate N-acetyltransferase (Asp-NAT), which is encoded by N-acetyltransferase 8-like (Nat8l), and has been implicated in the regulation of energy expenditure in BAT [24]. Nat8l expression in BAT increases upon cold acclimation and under nutrient-rich conditions [12,24], whereas it decreases during cold deacclimation and in obesity [12,25]. However, NAA remains the only Ac-AA with a well-established enzymatic origin in BAT.

Notably, cold acclimation has been shown to increase proteasomal activity in BAT [26], leading to the hypothesis that protein degradation may contribute to Ac-AA levels in BAT [10,12]. In contrast, ^13^C-labeling studies demonstrate that glucose-derived acetyl-CoA can serve as a direct precursor for Ac-AA formation [10,24]. Moreover, ^15^N-tracer studies in BAT showed that branched-chain amino acids (BCAAs) are directly incorporated into Ac-AAs, and that disruption of mitochondrial BCAA transport resulted in reduced Ac-AA levels [22,24]. While these findings suggest the presence of multiple contributing sources, they more importantly provide direct isotopic evidence for the de novo formation of Ac-AAs from acetyl-CoA and amino acids in BAT.

Several BAT studies have proposed potential physiological functions for Ac-AAs. In particular, Ac-AAs have been suggested to act as buffers for acetyl-CoA, either regulating protein acetylation or lipogenesis, or serving as a reversible sink for two-carbon units [10,12]. Beyond cellular effects, BAT-derived Ac-AAs have also been implicated in systemic processes, including their use as substrates for hepatic glutathione synthesis [22] and potential roles in inter-organ signaling [12]. However, despite these proposed functions, the metabolic origins and physiological functions of Ac-AAs in BAT remain unresolved, and multiple studies have explicitly noted the need for further mechanistic investigation [10,12,27]. The mechanistic plausibility and available evidence supporting these and other proposed physiological functions are critically discussed in later sections of this review.

### 2.3 Ac-AAs in disease and inborn errors of metabolism

As this review focuses primarily on physiological contexts, pathological conditions are addressed only briefly to provide contextual background. Ac-AAs are increasingly recognized as biomarkers across multiple pathological conditions. Inborn errors of metabolism characterized by elevated circulating amino acids have consistently been associated with Ac-AA accumulation. This is reflected by increased levels of N-acetylphenylalanine in phenylketonuria, N-acetyltyrosine in tyrosinemia, and N-acetylated BCAAs in maple syrup urine disease [4], as well as N-acetylhistidine in histidinemia [28] and N-acetylcitrulline in citrullinemia [29]. It has been suggested that in disorders characterized by amino acid accumulation, a fraction is converted into the corresponding N-acetylated derivatives [30]. However, the underlying physiological mechanisms have not been discussed explicitly.

Beyond that, altered levels of Ac-AAs have been reported across multiple cancer studies, particularly in response to drug treatment [5,[31], [32], [33]]. To explain these observations**,** it has been proposed that acetylation of free amino acids may modulate acetyl-CoA availability, thereby regulating protein acetylation and downstream gene expression, with potential implications for cancer metabolism [5,31]. However, despite multiple hypotheses, mechanistic studies remain scarce, and the origins and functions of Ac-AAs in cancer remain poorly defined.

Additionally, Ac-AAs are frequently reported in studies of kidney health and have been associated with CKD, reduced renal function, and kidney failure [3,[34], [35], [36], [37]]. Following glomerular filtration, circulating Ac-AAs are hydrolyzed in the kidneys by aminoacylases (see section Deacetylation and turnover of Ac-AAs). Accordingly, Ac-AAs are primarily regarded as markers of glomerular filtration and renal function rather than as having direct physiological relevance to the kidneys. However, previous studies have noted that the functional roles of Ac-AAs in kidney physiology remain incompletely understood [35,37].

Several studies have reported associations between Ac-AAs and metabolic health and related outcomes, including type 2 diabetes, overweight and obesity, and all-cause mortality [6,[38], [39], [40]]. Among disease-specific hypotheses, Ac-AAs have been proposed to modulate protein acetylation, with potential consequences for metabolic regulation [38,39]. However, across these diverse pathological conditions, alterations in Ac-AA levels may reflect heterogeneous and context-specific metabolic changes that differ from regulated physiological responses such as exercise or cold acclimation in BAT.

## 3 Degradation of N-terminally acetylated proteins

The presence of Ac-AAs is commonly attributed to the degradation of N-terminally acetylated proteins, which currently represents the best-characterized source of a broad spectrum of Ac-AAs. Protein acetylation is the transfer of an acetyl group from acetyl-CoA to either the ε-amino group of lysine residues or the α-amino group at the N-terminus of a polypeptide. Among these two acetylation modalities, only the degradation of N-terminally acetylated proteins can generate diverse Ac-AAs. In mammals, N-terminal protein acetylation is catalyzed by N-terminal acetyltransferases (NatA–NatF), with NatA, NatB, and NatC accounting for approximately 80% of N-terminal acetylation reactions in human cells. N-terminal acetylation commonly targets the initiator methionine, whereas cleavage of this residue results in acetylation of the subsequent amino acid. Accordingly, the first two amino acids of a polypeptide determine N-terminal acetylation and N-acetyltransferase specificity. For example, NatA preferentially acetylates N-termini containing small amino acids (e.g., Ala, Ser, Thr, and Gly) following initiator methionine removal, whereas NatB primarily targets Met–Asp, Met–Asn, Met–Glu, and Met–Gln N-termini, and NatC preferentially acetylates methionine-retaining N-termini followed by hydrophobic or amphipathic residues, including Leu, Val, and Lys [41,42].

Degradation of N-terminally acetylated proteins offers a mechanistically intuitive explanation for the presence of diverse Ac-AAs. However, the recurrence patterns of Ac-AAs across exercise and BAT metabolomics studies do not align with established N-terminal protein acetylation frequencies. Several of the reported Ac-AAs are predominantly acetylated by NatE and NatF, which target only a limited fraction of human proteins [41,42]. For instance, isoleucine, tryptophan, and tyrosine are quantitatively rare substrates for N-terminal acetylation, yet acetylated derivatives of these amino acids have been repeatedly reported across exercise and BAT metabolomics studies. In contrast, amino acids that are frequently N-terminally acetylated, including methionine and alanine, are not consistently observed across these datasets. Despite the role of methionine as the initiator amino acid in most human proteins, N-acetylmethionine exhibits inconsistent directions of change across studies. However, proline is the only amino acid not known to undergo N-terminal acetylation [41,42], and accordingly, we did not identify any exercise or BAT metabolomics studies reporting increases in N-acetylproline. Notably, non-proteinogenic N-acetylated derivatives cannot be attributed to protein degradation, as the corresponding amino acids are not constituents of proteins.

Moreover, attributing the observed increases in Ac-AAs solely to protein degradation would require a substantial upregulation of proteolysis. While increased proteasomal activity has been reported during BAT acclimation [26], available evidence suggests that protein degradation is not increased during exercise [43,44]. In addition, alterations in Ac-AAs have been reported after relatively short exercise durations, a time frame unlikely to permit substantial protein degradation. Quantitatively, BAT acclimation has been shown to increase Ac-AA levels by up to 4,000-fold compared with circulating levels [10], a magnitude that appears unlikely to originate solely from protein degradation. Additionally, circulating free acetyllysine levels do not correlate with the acetyllysine content of circulating proteins in humans [36], suggesting a potential decoupling between acetylated proteins and Ac-AAs. Collectively, protein degradation contributes to the systemic pool of Ac-AAs but is unlikely to fully account for the observed alterations in exercise and BAT datasets, suggesting the involvement of additional or complementary mechanisms.

## 4 Determinants of Ac-AA formation

### 4.1 Ac-AAs reflect intracellular acetyl-CoA and amino acid availability

Ac-AAs are predominantly observed in response to high-intensity exercise and cold acclimation in BAT and, upon recovery from exercise or during BAT deacclimation, they decline toward baseline levels. High-intensity exercise and cold exposure in BAT both represent states of high metabolic activity, characterized by increased mitochondrial flux to meet the energetic demands of muscular work and adaptive thermogenesis. These conditions are accompanied by increased availability of cellular acetyl-CoA. ^13^C-labeling studies demonstrate that glucose-derived acetyl-CoA can serve as a direct precursor for Ac-AA formation [10,24]. Increases in Ac-AA levels have also been observed following protein or BCAA supplementation [22,45], as well as in metabolic diseases characterized by elevated circulating free amino acids [4,28,29]. Notably, ^15^N tracer studies have shown that Ac-AAs are directly synthesized from free amino acids, and that disruption of mitochondrial BCAA transport results in reduced Ac-AA levels [22,24]. Collectively, these findings demonstrate that Ac-AA formation is driven by conditions of elevated intracellular acetyl-CoA and amino acid availability.

### 4.2 Tissue and subcellular origins of Ac-AA formation

BAT has been identified as a major producer of Ac-AAs during cold exposure. While some Ac-AAs have been shown to be exchanged between BAT and the circulation [23], it remains unclear which Ac-AAs reach the circulation and to what extent, as certain Ac-AAs that accumulate in BAT have not been detected in serum during cold exposure, and the relative abundances of those that are detected are often markedly lower than in BAT [10]. During exercise, skeletal muscle is likely an important contributor to circulating Ac-AAs due to the substantial increase in metabolic flux and intracellular acetyl-CoA availability. However, most exercise studies have assessed Ac-AAs exclusively in the circulation. Therefore, it remains unclear whether other metabolically active tissues, such as the heart, liver, or BAT, also contribute to circulating Ac-AA levels during exercise or primarily take up Ac-AAs generated elsewhere. Importantly, differences in tissue mass should be considered when interpreting potential contributions to circulating Ac-AA levels, as skeletal muscle constitutes a substantially larger proportion of total body mass than BAT and other metabolically active organs.

While Ac-AA formation may occur in several cellular compartments, a mitochondrial origin appears most plausible, given the substantial increase in mitochondrial acetyl-CoA availability during conditions of high metabolic activity. In addition to acetyl-CoA, mitochondria also contain amino acid pools that could serve as substrates for Ac-AA formation. In line with this, NAA and NAG have both been shown to be synthesized within mitochondria in their respective tissues [8,9]. Moreover, Verkerke et al. demonstrated that mice lacking the mitochondrial BCAA carrier (MBC) exhibited reduced NAA and NAG levels in BAT [22]. Consistently, Huber et al. used a ^13^C-labeling approach to demonstrate that glucose can contribute to both the acetyl and amino acid moieties of NAA in BAT [24], suggesting formation from mitochondrially derived substrates rather than transport of both precursors to other cellular compartments prior to acetylation. However, NAA currently represents the best-characterized Ac-AA in BAT, and whether these findings are representative of other Ac-AAs remains unknown. Collectively, the available evidence suggests a mitochondrial origin of Ac-AA formation within highly metabolically active tissues.

### 4.3 Substrates of Ac-AA formation

Glucose-derived acetyl-CoA has been demonstrated to serve as a precursor for Ac-AA formation [10,24]. While fatty acid oxidation represents another major source of acetyl-CoA and is therefore likely to contribute to Ac-AA formation, this has not yet been demonstrated experimentally. In contrast, stable isotope tracing in BAT indicated only a minor contribution of acetate to the acetyl moiety of NAA, whereas the carbon contribution of BCAAs and glutamine was also minimal [24]. These findings are consistent with the relative contributions of these substrates to cellular acetyl-CoA availability, with glucose and fatty acids generally contributing more substantially than acetate and amino acids [46]. These observations support the hypothesis that the relative contributions of different substrates to Ac-AA formation broadly reflect their contributions to cellular acetyl-CoA availability, although direct evidence for fatty acid-derived acetyl-CoA and for Ac-AAs beyond NAA remains lacking.

Across exercise and BAT metabolomics studies, certain Ac-AAs (e.g. Ac-Glu, Ac-Met, Ac-Leu) are consistently reported across multiple studies and tissues, whereas others (e.g. Ac-Cys, Ac-Pro, Ac-Lys, Ac-His) appear only sporadically. Notably, this pattern does not appear to be explained by amino acid–specific physicochemical properties such as size, polarity, or charge. Similarly, reported plasma concentrations of the corresponding free amino acids [47] do not align with the observed detection frequency of Ac-AAs. However, the recurrence of Ac-AAs may relate to intracellular concentrations of the corresponding free amino acids, as the most abundant muscular amino acids, glutamine and glutamate [48], are also among the most frequently observed Ac-AAs. Likewise, cysteine, proline, histidine, and tryptophan have been reported among the least abundant amino acids in rat liver mitochondria and are also among the least frequently observed Ac-AAs [49]. However, whether these observations extend to human skeletal muscle and BAT mitochondria remains unknown. The inconsistent directions of change observed for certain Ac-AAs across studies may reflect amino acid–specific metabolic properties. For instance, N-acetylmethionine and N-acetyllysine are closely linked to protein acetylation, and they may therefore partially reflect protein acetylation dynamics rather than other potential metabolic processes. NAA has been consistently reported to increase in cold-exposed BAT but to decrease following exercise, which may potentially reflect tissue-specific NAA metabolism. Taken together, no clear patterns emerge linking Ac-AAs to characteristics of the corresponding free amino acids, such as circulating concentrations or physicochemical properties, although intracellular amino acid abundance may represent one factor contributing to the observed acetylation patterns.

### 4.4 Cellular transport of Ac-AAs

The detection of Ac-AAs in the circulation and across multiple tissues indicates that Ac-AAs are capable of crossing cellular membranes. However, no dedicated transporters have been identified that facilitate the transport of Ac-AAs across the plasma membrane, the intestinal epithelium, or the mitochondrial membranes. Despite the considerable interest in NAA and NAG owing to their well-established physiological functions, the mechanisms governing their transport across cellular membranes remain incompletely understood [8,50]. The strongest candidate for NAA transport is the sodium-dependent dicarboxylate transporter NaDC3, which appears to mediate NAA transport within the brain and may also facilitate renal uptake [8]. While dicarboxylate transporters may mediate the transport of selected dicarboxylic Ac-AAs, the considerable structural diversity of Ac-AAs suggests that additional transport systems are required. Similarly, the transport of amino acids across cellular membranes is mediated by a wide variety of transport systems [51]. Notably, amino acid transport across the intestinal epithelium, plasma membrane, and mitochondrial membranes is mediated by distinct transporter systems, suggesting that Ac-AA transport may involve multiple transporter families depending on the tissue and cellular compartment. Given the broad substrate specificity of some amino acid transporters toward modified amino acids [52,53], it is conceivable that Ac-AAs are transported by existing amino acid transporters. However, the transport systems that contribute to cellular and subcellular Ac-AA transport remain largely unexplored and warrant further investigation.

## 5 Mechanisms of Ac-AA formation

### 5.1 Substrate-driven formation

Changes in cellular Ac-AA concentrations in response to physiological stimuli do not necessarily imply active regulation of Ac-AA synthesis. The simultaneous increase of a broad spectrum of structurally diverse Ac-AAs across diverse physiological conditions suggests a generalized response to elevated substrate availability. Consistent with this, alterations in Ac-AA levels often follow changes in the availability of their precursor amino acids and acetyl-CoA-generating substrates. Therefore, increased availability of acetyl-CoA and amino acids may be sufficient to promote Ac-AA formation through mass-action kinetics, irrespective of whether acetylation occurs via enzymatic or non-enzymatic mechanisms. The observation that some Ac-AAs accumulate in BAT without a corresponding increase in the circulation [10], suggests that a proportion of Ac-AAs may undergo intracellular deacetylation rather than being released into the circulation. Such a mechanism would be consistent with a reversible, mass-action-driven model of Ac-AA formation in which Ac-AAs are synthesized during periods of elevated substrate availability and subsequently deacetylated as substrate concentrations decline. Taken together, these observations support the hypothesis that substrate-driven mass-action kinetics represent a central mechanism underlying Ac-AA formation.

### 5.2 Enzymatic acetylation

While substrate-driven mass-action kinetics may determine the extent of Ac-AA formation, the acetylation reaction itself may proceed through different mechanisms. Acetylation of free amino acids represents a possible mechanism for Ac-AA formation, as mammalian acetyltransferases catalyze acetyl-CoA–dependent transfer of acetyl groups to a wide range of substrates. Several N-acetyltransferases (NATs) have been shown to catalyze the acetylation of specific free amino acids, including aspartate N-acetyltransferase [54] and glutamate N-acetyltransferase [55]. However, acetyltransferases with broad specificity toward multiple free amino acids remain poorly characterized. NATs that are primarily expressed in the liver and kidneys and traditionally involved in xenobiotic metabolism may be relevant, as emerging evidence suggests that certain members may contribute to Ac-AA formation. For instance, genetic variants in the NAT8 gene have been associated with circulating levels of multiple Ac-AAs [35]. Moreover, in vitro studies suggest that ACY1, the enzyme responsible for the deacetylation of Ac-AAs, may also catalyze the synthesis of Ac-AAs under favorable reaction conditions [56]. Additionally, growing evidence suggests that several NAT isoforms may display broader substrate specificity than previously recognized [57]. However, given their largely tissue-restricted expression, these NATs are unlikely to account for the broad spectrum of Ac-AAs detected across multiple tissues, including skeletal muscle and BAT. NAT1 represents an example of a widely expressed acetyltransferase, suggesting that its functions extend beyond xenobiotic metabolism [58], although its relevance to Ac-AA metabolism remains unclear. Widely expressed acetyltransferases, including those involved in protein acetylation or the acetylation of specific free amino acids, may represent plausible enzymes for future Ac-AA research. However, currently characterized acetyltransferases do not exhibit the broad substrate specificity and widespread tissue expression required to explain the systemic formation of Ac-AAs.

### 5.3 Non-enzymatic acetylation

Given the high reactivity of acetyl-CoA and the mildly alkaline mitochondrial matrix, non-enzymatic acetylation represents another plausible mechanism of Ac-AA formation. Such a mechanism would also be compatible with the proposed substrate-driven formation, as increasing substrate concentrations would be expected to increase the probability of spontaneous acetylation reactions. Similar processes are well documented under conditions of elevated acetyl-CoA availability. For instance, non-enzymatic acetylation of protein lysine residues is recognized as a widespread protein modification [59,60]. Notably, non-enzymatic acetylation of free amino acids has been demonstrated in vitro, such as the formation of N-acetyltaurine through condensation of acetyl-CoA with taurine [13], and the non-enzymatic formation of N-acetylalanine from pyruvate and ammonia [61,62]. However, current knowledge primarily derives from studies focusing on individual Ac-AAs and from in vitro experiments. Therefore, the substrate concentrations required to permit non-enzymatic acetylation across a broad spectrum of amino acids remain uncertain, and it is unclear whether such reactions occur under physiological conditions or in which tissues they may be relevant. Together, these observations support non-enzymatic acetylation as a chemically plausible mechanism for Ac-AA formation, while highlighting that its physiological relevance and tissue specificity remain to be defined.

Together, these observations suggest that Ac-AA formation may be largely governed by substrate-driven, mass-action kinetics, irrespective of whether acetylation proceeds through enzymatic or non-enzymatic mechanisms. While currently characterized acetyltransferases do not exhibit the broad substrate specificity and widespread tissue expression required to fully explain the systemic formation of Ac-AAs, non-enzymatic acetylation represents a chemically plausible alternative, although its physiological relevance in vivo remains to be established.

### 5.4 Gut microbial synthesis

It has been demonstrated that Ac-AAs can be synthesized in the gut, likely mediated by bacterial enzymatic activity within the intestinal microbiota [13,63,64]. In line with this, some circulating Ac-AAs have been reported to correlate with fecal levels of their corresponding amino acids [19]. Moreover, initial evidence suggests that increased dietary fibre intake is associated with elevated Ac-AA levels [63]. However, the precise mechanisms and conditions underlying intestinal Ac-AA synthesis remain insufficiently understood, and the rapid changes of Ac-AA levels, together with their presence in multiple metabolically active tissues, suggests that gut microbial activity is insufficient to fully account for their systemic alterations. Nevertheless, investigating the mechanisms underlying microbial Ac-AA synthesis may provide insight into analogous acetylation pathways in mammalian tissues. Importantly, the proposed mechanisms are not mutually exclusive, and multiple pathways may operate concurrently.

## 6 Deacetylation and turnover of Ac-AAs

**Figure 2:**
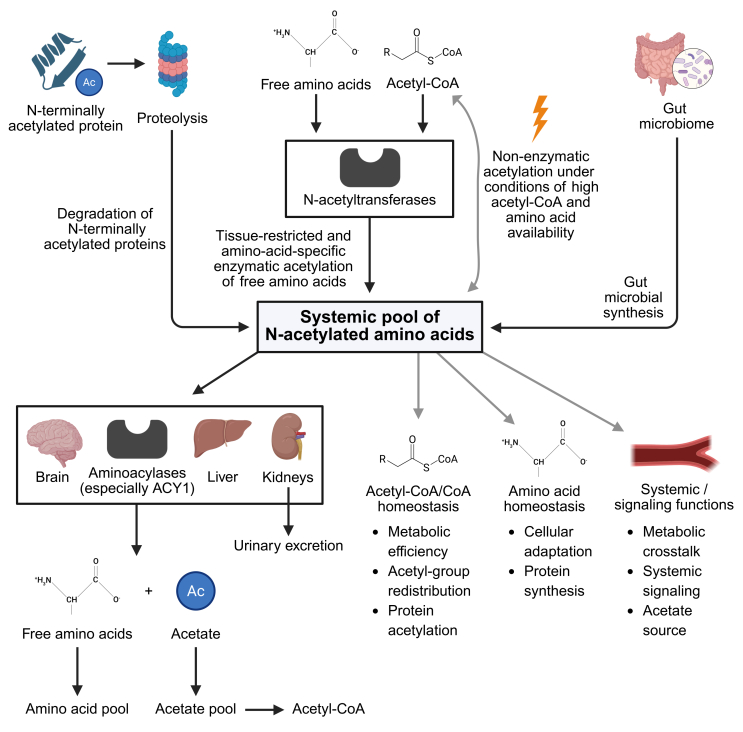
**Systemic origins and physiological functions of N-acetylated amino acids.** Black arrows indicate metabolic processes demonstrated in vivo, while grey arrows denote indicated pathways and functions.

The proposed degradation pathway of Ac-AAs is illustrated in Figure 2. Ac-AAs undergo glomerular filtration in the kidneys, and a small fraction is typically excreted in the urine [35,37]. However, the majority of circulating Ac-AAs can be deacetylated by aminoacylases (ACY1, ACY2, and ACY3), predominantly expressed in the kidneys, liver, and brain [65]. Aminoacylases catalyze the hydrolytic cleavage of Ac-AAs into their corresponding free amino acids and acetate. Aminoacylase 1 (ACY1) preferentially hydrolyzes neutral aliphatic Ac-AAs, aminoacylase 2 (ACY2) is highly specific for N-acetylaspartate (NAA), and aminoacylase 3 (ACY3) primarily hydrolyzes aromatic Ac-AAs [66]. In addition to aminoacylases, other enzymes may contribute to the deacetylation of Ac-AAs, such as the phosphotriesterase-related protein (PTER), a physiological N-acetyltaurine hydrolase, which has been reported to exhibit modest hydrolysis activity toward Ac-AAs [13].

Changes in Ac-AA levels in BAT coincide with altered aminoacylase expression, which decreases during cold acclimation and increases upon deacclimation [12]. Due to its relatively broad substrate specificity, ACY1 has been the most extensively studied member of the aminoacylase family. In cases of ACY1 deficiency resulting from rare genetic mutations, circulating levels and urinary excretion of Ac-AAs increase [65], and patients present with a broad spectrum of physiological disorders [30,67]. Moreover, several studies support a tumor-suppressive role of ACY1 [68,69], and a potential relationship between Ac-AAs and this function has been hypothesized [5]. Recently, a conference report described associations between ACY1 and metabolic abnormalities in mice, including increased body weight and impaired glycemic control, and proposed ACY1 as a previously unrecognized regulator of metabolic homeostasis [70]. Collectively, these findings highlight the role of aminoacylases, especially ACY1, in Ac-AA metabolism and suggest that aminoacylases may contribute to the reported associations of Ac-AAs with various diseases, although further mechanistic research is required.

## 7 Magnitude of Ac-AA accumulation

Quantitative interpretation of Ac-AA concentrations remains challenging, as most metabolomics studies report relative abundances rather than absolute concentrations. To our knowledge, only Pileggi et al. reported absolute concentrations of Ac-AAs [12]. In that study, the 11 quantified Ac-AAs exhibited a cumulative concentration difference of approximately 0.6 μmol/g BAT between 6 weeks of cold acclimation and 48 h of thermoneutrality, calculated as the sum of the differences in mean concentrations across the quantified Ac-AAs reported in Supplementary Data S2. However, only 11 Ac-AAs were quantified, and acetylated derivatives of non-proteinogenic amino acids, some of which are highly abundant in mammalian tissues [48], were not assessed. Furthermore, intracellular Ac-AA concentrations may not directly reflect Ac-AA formation rates, as Ac-AAs are partly released into the circulation, and concentrations may differ substantially between intracellular compartments. Consequently, the cumulative difference likely represents only a partial estimate of the total Ac-AA response to cold acclimation. Moreover, reported fold changes of individual Ac-AAs appear to vary considerably across BAT studies, ranging from modest increases to several-fold elevations depending on the specific Ac-AA [10,12]. While fold changes in response to exercise appear broadly comparable to those observed during BAT acclimation, most exercise studies report circulating rather than tissue Ac-AA levels, and the absence of absolute concentration data precludes direct comparison of Ac-AA concentration changes in response to exercise. Taken together, the available evidence indicates that Ac-AA concentrations in BAT reach at least the low μmol/g range, although comprehensive quantitative measurements during acute exercise and BAT acclimation remain scarce.

To place Ac-AA concentrations into context, they can be compared with the concentrations and dynamics of intracellular acetyl-CoA, coenzyme A (CoASH), and free amino acids. As absolute Ac-AA concentrations are currently available only for BAT, comparisons with exercising skeletal muscle should be considered indirect. Although reported values vary between tissues and experimental conditions, acetyl-CoA and CoA concentrations in cold-acclimated BAT and exercising skeletal muscle generally remain below approximately 0.3 μmol/g tissue [12,71]. However, the metabolic relevance of acetyl-CoA is determined not only by its pool size but also by its exceptionally high flux through metabolic pathways. While direct measurements of acetyl-CoA flux are challenging, it can be approximated from pyruvate dehydrogenase complex (PDC) activity. Studies of high-intensity exercise typically report PDC activities of approximately 1.2 μmol g^−1^ tissue·min^−1^ [72,73], which is broadly consistent with reported PDC activities in cold-acclimated BAT [74]. Although PDC activity does not capture all sources of acetyl-CoA production, it provides a useful approximation of the overall magnitude of acetyl-CoA flux. These observations suggest that Ac-AAs can accumulate to concentrations that exceed intracellular acetyl-CoA pools, while acetyl-CoA flux occurs at rates substantially greater than the size of the acetyl-CoA pool itself. However, these values should be interpreted primarily as providing context regarding the order of magnitude of the respective metabolite concentrations rather than as precise quantitative estimates.

Intracellular free amino acid concentrations in skeletal muscle have been reported at approximately 34.5 mmol/L intracellular water [48], corresponding to approximately 23 μmol/g tissue based on the reported tissue water content. However, glutamine alone accounts for the majority of this concentration and excluding glutamine reduces the combined amino acid pool to approximately 9.0 μmol/g tissue. In rat BAT, the combined concentration of free proteinogenic amino acids has been reported at approximately 17.9 mM [75], corresponding to approximately 9.8 μmol/g tissue based on the reported tissue water content. Importantly, intracellular amino acid pools are distributed across multiple subcellular compartments, including the mitochondria, cytosol, and nucleus. Consequently, amino acid concentrations within individual subcellular compartments, such as the mitochondrial matrix, may differ substantially from those estimated from whole-tissue measurements. Moreover, amino acids undergo bidirectional exchange with the circulation, and changes in metabolic flux may influence amino acid metabolism, as several amino acids are synthesized from TCA cycle intermediates. Collectively, these estimates suggest that Ac-AAs may represent a considerable fraction of free proteinogenic amino acid pools during BAT acclimation and potentially during acute exercise. However, the available data remain insufficient for a precise quantitative comparison across tissues, subcellular compartments, and physiological conditions.

## 8 Potential physiological functions of Ac-AAs

### 8.1 Ac-AAs as modulators of intracellular acetyl-CoA and CoASH homeostasis

The potential physiological functions of Ac-AAs are illustrated in Figure 2. As outlined above, Ac-AA levels increase under conditions of elevated intracellular acetyl-CoA availability. Intracellular acetyl-CoA and free CoASH availability are central regulators of cellular processes, including metabolic enzyme activity, acetylation reactions, and cellular signaling [76]. Accordingly, cells employ multiple mechanisms to regulate acetyl-CoA and CoASH levels and thereby the acetyl-CoA/CoA ratio. For instance, acetyl-CoA exerts feedback regulation of mitochondrial pyruvate metabolism by inhibiting pyruvate dehydrogenase while activating pyruvate carboxylase [7,76]. One of the best-characterized mechanisms regulating intracellular acetyl-CoA and CoASH homeostasis involves l-carnitine, which reversibly accepts acetyl groups from acetyl-CoA, generating acetylcarnitine and thereby modulating the intracellular acetyl-CoA/CoA balance [77]. A similar mechanism has been described for taurine, which can undergo reversible acetylation to form N-acetyltaurine [78,79]. Moreover, acetyl groups can be temporarily stored in macromolecular structures such as histones, which may function as reversible reservoirs of acetyl groups [80,81]. These observations demonstrate that cells employ multiple molecular mechanisms to modulate intracellular acetyl-CoA and CoASH availability.

Although acetyl-CoA flux during high-intensity exercise and BAT acclimation is estimated to be around 1.2 μmol g^−1^ tissue·min^−1^, acetyl-CoA is continuously consumed by the TCA cycle and can also supply acetyl groups for lipid synthesis and acetylation reactions in other cellular compartments [7]. Therefore, even a small fraction of total acetyl-CoA flux directed toward Ac-AA formation could influence intracellular acetyl-CoA and CoASH availability. During high-intensity exercise, free carnitine concentrations in skeletal muscle decrease from approximately 4.7 to 1.4 μmol/g tissue, whereas acetylcarnitine concentrations increase from approximately 0.7 to 3.9 μmol/g tissue (all values were estimated from reported dry-weight measurements assuming a skeletal muscle water content of 75%) [71]. Notably, these concentrations are broadly comparable to the low μmol/g concentrations estimated for Ac-AAs in BAT. The majority of the free carnitine pool is converted to acetylcarnitine within as little as 3 min of the onset of high-intensity exercise [71]. Early temporal dynamics of Ac-AA formation remain unexplored, as the earliest measurements were obtained after 30 min of exercise and hours to weeks after cold acclimation in BAT. Using isotope tracing, Ac-AA formation was detected as early as 15 min after glucose gavage, although earlier time points were not examined [10]. As acetylcarnitine formation is thought to be particularly important during the rapid increase in acetyl-CoA flux at exercise onset, Ac-AA formation may also be especially relevant during this transient period. Collectively, these observations support the hypothesis that acetylation of free amino acids may contribute to the modulation of intracellular acetyl-CoA and CoASH availability alongside established regulatory mechanisms.

Importantly, any transfer of acetyl groups from acetyl-CoA to free amino acids would inherently influence intracellular acetyl-CoA and CoASH availability, even in the absence of specific regulatory control. Given that free amino acid pools exceed the free carnitine pool, acetylation of free amino acids may provide an additional mechanism influencing intracellular acetyl-CoA and CoASH availability, particularly under conditions of elevated acetyl-CoA availability and reduced free carnitine concentrations. Although a role in intracellular acetyl-CoA and CoASH homeostasis appears plausible, supporting evidence remains largely indirect, as direct measurements of Ac-AA concentrations are scarce, particularly in response to acute exercise. However, the contribution of Ac-AAs to acetyl-CoA homeostasis could be investigated using a short-duration, high-intensity exercise intervention combined with targeted metabolomic analysis of skeletal muscle biopsy samples.

### 8.2 Metabolic consequences of Ac-AA formation for acetyl-CoA and CoASH homeostasis

Ac-AA formation may influence cellular function through multiple mechanisms. For instance, Ac-AA formation could support cellular energy metabolism by limiting accumulation of acetyl-CoA and elevations in the acetyl-CoA/CoA ratio. Reducing acetyl-CoA concentrations and the acetyl-CoA/CoA ratio decreases feedback inhibition of PDC, thereby promoting glucose oxidation and overall metabolic flux, a well-established consequence of l-carnitine–mediated acetyl-group buffering [77,82]. In parallel, transfer of acetyl groups from acetyl-CoA regenerates free CoASH, preventing CoASH availability from becoming rate limiting for CoA-dependent pathways during periods of high acetyl-CoA flux. In fact, Constantin-Teodosiu et al. argued that, at maximal PDC activity during high-intensity exercise (estimated at 1.2–1.5 μmol g^−1^ tissue·min^−1^), the resting intramuscular CoASH pool would become fully acetylated within approximately 1 s if no mechanism existed to rapidly regenerate free CoASH [73,83]. Accordingly, acetyl-group buffering has been shown to be particularly important during the rapid increase in acetyl-CoA flux immediately after exercise onset, where acetylcarnitine formation promotes overall metabolic efficiency by maintaining free CoASH availability [83]. As metabolic flux declines, acetyl groups stored in acetylcarnitine can be transferred back to CoASH to regenerate acetyl-CoA [83], a principle that may also apply to Ac-AAs if their formation is governed primarily by mass-action kinetics. Acetyl groups could also be transported across cellular membranes and delivered to other cellular compartments, where they could potentially contribute to processes requiring acetyl-CoA, such as lipid synthesis or acetylation reactions. Thus, sequestration of acetyl groups into Ac-AAs may represent a previously unrecognized mechanism for storing excess acetyl groups while regenerating free CoASH, thereby maintaining substrate flux through glucose and fatty acid metabolism when acetyl-CoA production exceeds the capacity of the TCA cycle. This mechanism could be particularly relevant during high-intensity exercise and BAT thermogenesis, where maintaining substrate flux under conditions of rapid acetyl-CoA generation may help sustain exercise capacity and thermogenic function.

Ac-AA formation may have implications beyond energy metabolism and influence cellular acetylation processes. Protein acetylation is tightly coupled to intracellular acetyl-CoA availability [84] and represents a reversible modification that regulates diverse cellular functions. For instance, modulation of cellular histone acetylation has been recognized as a key regulator of genes involved in metabolic control [85], with potential implications for metabolic efficiency during exercise and BAT acclimation. Evidence suggests that acetylation patterns can change within minutes in response to altered cellular conditions [86], and both histone and non-histone protein acetylation have been shown to increase in response to exercise, due to elevated intracellular acetyl-CoA availability [87,88]. Consequently, mechanisms that regulate cellular acetyl-CoA availability can modulate acetylation capacity, with broad implications for metabolic function [76]. Notably, this regulatory concept has been demonstrated for l-carnitine, which modulates the cellular acetylproteome through its effects on intracellular acetyl-CoA availability [60]. This mechanism operates not only in mitochondria but also in other cellular compartments, including the nucleus, where mitochondria-derived acetylcarnitine has been implicated in regulation of the acetylproteome [89]. Similarly, NAA has been shown to modulate cytoplasmic acetyl-CoA availability, thereby influencing histone acetylation and gene expression in BAT [90], demonstrating that at least specific Ac-AAs can participate in the regulation of cellular acetylation processes. These regulatory effects are likely not restricted to l-carnitine and NAA but may extend to other mechanisms that regulate intracellular acetyl-CoA availability, potentially including the formation of Ac-AAs. As Ac-AAs may occur at substantial intracellular concentrations and are released into the circulation, they may also influence systemic acetyl-group availability and thereby modulate acetylation capacity across tissues.

Importantly, acetylation of free amino acids does not result in irreversible loss of acetyl groups or amino acids. Assuming that Ac-AA formation is governed by mass-action kinetics, changes in substrate and product concentrations could favor not only Ac-AA formation but also the conversion of Ac-AAs back to free amino acids. Alternatively, circulating Ac-AAs can be hydrolyzed by aminoacylases, releasing acetate that can subsequently be converted back to acetyl-CoA, thereby enabling temporary redistribution of acetyl groups. Conceptually, this resembles metabolic shuttle systems, such as the lactate shuttle, in which intermediates are transiently exported to sustain metabolic flux while remaining recoverable. Accordingly, Ac-AA formation may permit temporary storage and redistribution of acetyl groups and amino acids while preserving their metabolic availability.

### 8.3 Ac-AAs as modulators of intracellular amino acid homeostasis

The magnitude of estimated intracellular Ac-AA concentrations relative to cellular amino acid pools suggests that Ac-AA formation could represent a considerable component of cellular amino acid homeostasis. Amino acids serve diverse cellular functions, acting as substrates for energy metabolism, protein synthesis, and the biosynthesis of bioactive molecules, while also regulating cellular signaling and gene expression [91]. Fluctuations in amino acid uptake, synthesis, utilization, and protein turnover can result in transient increases in intracellular amino acid availability [92], which can disrupt cellular homeostasis and have been associated with a range of adverse metabolic effects [92,93]. Accordingly, cells employ multiple mechanisms to maintain cellular amino acid homeostasis, including the regulation of amino acid transport across cellular membranes and the sequestration of excess amino acids into intracellular compartments [93,94]. Ac-AA formation may represent a complementary mechanism for limiting excessive intracellular amino acid accumulation through the transient sequestration of free amino acids. This concept is consistent with the observed accumulation of specific Ac-AAs in metabolic diseases characterized by elevated levels of their corresponding amino acids. Because specific amino acids are key regulators of mTOR signaling and autophagy [95,96], the transient sequestration of free amino acids into Ac-AAs may influence the balance between anabolic and catabolic cellular processes. In addition to mTOR signaling, amino acid availability regulates cellular responses through tRNA-charging–dependent sensing pathways [97], suggesting that Ac-AA formation may influence these processes by altering the intracellular availability of free amino acids. Through effects on mTOR signaling and tRNA-dependent nutrient-sensing pathways, Ac-AA formation may influence protein synthesis and broader translational programs. Consequently, Ac-AA formation may have broader physiological effects by linking amino acid homeostasis to the regulation of adaptive cellular responses. However, intracellular amino acid availability is highly dynamic, and the quantitative contribution of Ac-AA formation remains incompletely understood.

### 8.4 Ac-AAs as systemic metabolites and signaling molecules

The detection of Ac-AAs in the circulation and in multiple tissues suggests relevance beyond local cellular metabolism and has prompted speculation about potential signaling functions and inter-organ communication [12,98]. As many established exerkines are closely linked to central metabolic intermediates, compounds originating from the key metabolic intermediate acetyl-CoA represent plausible candidates for studying signaling functions in exerkine research. In fact, numerous metabolites formed by the conjugation of amino acids with other metabolic intermediates have been shown to exert important signaling functions [99,100]. Consistent with this, N-acetyltaurine was recently identified as a signaling molecule influencing food intake and obesity, with its levels regulated by the hydrolase PTER [13]. Despite modest in vitro hydrolysis activity toward several Ac-AAs, genetic PTER deficiency did not result in broad changes in Ac-AA levels. However, the enzymatic relationship between PTER and N-acetyltaurine mirrors that between ACY1 and Ac-AAs, particularly considering that both ACY1 and Ac-AAs have been linked to metabolic diseases.

Reported concentrations of Ac-AAs in cold-exposed BAT, reaching the μmol/g tissue range, suggest that their hydrolysis could represent a substantial source of circulating acetate (∼181 ± 28 μmol L^−1^ under fasted conditions) [101]. Given that endogenous acetate production remains incompletely understood [102], Ac-AAs may represent a previously unrecognized contributor to circulating acetate levels. This may be metabolically relevant, as acetate has been proposed to act as a regulatory intermediate supporting acetyl-CoA availability in both the mitochondria and cytosol [102]. Moreover, acetate has been implicated in immune regulation [103] and may be linked to protein acetylation, as certain acetyltransferases have been shown to exhibit flexibility in substrate utilization [85]. Together, these observations suggest that hydrolysis of Ac-AAs may contribute substantially to systemic acetate metabolism and thereby influence acetyl-CoA availability.

Ac-AAs may be particularly relevant in the brain, as ACY1 is highly expressed in neural tissue [65] and Ac-AAs have been detected in brain tissues such as the hypothalamus [2]. Huppke–Brendel syndrome, a neurodevelopmental disorder caused by deficiency of the acetyl-CoA transporter 1 (AT-1), has been linked to decreased Ac-AA levels in cerebrospinal fluid. As AT-1 regulates the influx of acetyl-CoA from the cytosol into the ER lumen, an influence on protein acetylation was hypothesized, but the observed effects on protein acetylation were minor [104]. More recently, studies in rats have suggested a link between Ac-AAs and energy metabolism in the brain [105]. However, the precise physiological roles of Ac-AAs in the brain remain incompletely understood.

Beyond the well-established functions of NAA and NAG, evidence suggests broader physiological roles for individual Ac-AAs. For instance, recent research indicates that some Ac-AAs can serve as substrates supporting glutathione synthesis in the liver [22]. Moreover, N-acetylleucine has shown promising results as a potential therapeutic option in traumatic brain injury [106,107]. Generally, Ac-AAs are often observed individually and associated with diverse physiological and pathological outcomes. However, their individual physiological roles most often remain speculative.

## 9 Future research

The proposed physiological functions of Ac-AAs are plausible, particularly given their role in acetyl-CoA and amino acid metabolism, and are consistent with observations from related metabolites with similar biochemical roles. However, direct functional evidence remains sparse, and addressing this gap will be essential to determine whether Ac-AAs serve a regulatory role or solely reflect underlying acetyl group flux. While several potential origins of Ac-AAs have been proposed, their physiological relevance and relative contributions remain incompletely understood. Acetyltransferases with broad substrate specificity and tissue distribution have not been clearly identified, and the in vivo relevance of non-enzymatic acetylation remains uncertain. Direct experimental validation of the proposed mass-action model and the reversibility of Ac-AA formation will be essential to establish its physiological relevance. Likewise, the mechanisms governing tissue-specific production, release, uptake, and inter-organ trafficking of Ac-AAs remain incompletely understood.

Current evidence on Ac-AAs derives from studies across diverse species, tissue types, and physiological conditions. However, only a subset of exercise and BAT metabolomics studies report Ac-AAs, likely reflecting methodological constraints that limit their detectability. Specifically, detection may depend on physiological conditions, such as exercise performed at sufficiently high intensity. Moreover, Ac-AAs are mostly reported as byproducts of exploratory metabolomics analyses, while targeted investigations of Ac-AAs remain scarce. Consequently, absolute concentration changes in response to physiological stimuli remain poorly characterized, particularly in the context of exercise. However, such quantitative data are essential for understanding the physiological functions of Ac-AAs. In BAT research, evidence currently derives largely from mouse models, as validation in humans remains technically challenging. In addition, sex-specific effects may influence Ac-AA levels, as indicated in mouse models [17], yet most human studies to date have predominantly included male participants. Moreover, the pathological implications of altered Ac-AA metabolism across diverse diseases remain poorly understood and warrant further investigation. ACY1 represents a particularly interesting target for future research, given its central role in Ac-AA metabolism and its associations with diverse diseases.

## 10 Conclusions

N-acetylated amino acids consistently increase in response to high-intensity exercise and cold acclimation in BAT, and decline upon recovery and deacclimation. While degradation of N-terminally acetylated proteins and gut microbial synthesis contribute to the systemic pool of Ac-AAs, the temporal dynamics, quantitative magnitudes, and recurrence patterns observed across studies indicate that these mechanisms are insufficient to explain the reported alterations in Ac-AA levels. Instead, several lines of evidence support a mechanistic framework in which Ac-AA formation increases under conditions of elevated intracellular acetyl-CoA and amino acid availability. Based on current evidence, Ac-AA formation may be most relevant in the mitochondria of highly metabolically active tissues. Both enzymatic and non-enzymatic acetylation are plausible mechanisms for Ac-AA formation, potentially driven by mass-action kinetics, but their physiological relevance in vivo remains insufficiently understood. Following their formation, Ac-AAs can enter the circulation and are transported to organs such as the liver and kidneys, where aminoacylases hydrolyze them to amino acids and acetate, thereby enabling the recycling of acetyl groups and amino acids. Ac-AAs may therefore function as metabolic buffers by temporarily storing acetyl groups, regenerating free CoASH and modulating intracellular amino acid pools. By maintaining acetyl-CoA, CoASH, and amino acid homeostasis, Ac-AAs may support metabolic efficiency while influencing protein acetylation and gene expression. Available evidence also suggests potential systemic functions and signaling effects. Moreover, Ac-AAs have been implicated in various pathological conditions such as cancer, chronic kidney disease, and metabolic disorders, yet their role in these diseases remains poorly understood. ACY1 may provide a promising target for investigating their regulation and pathological effects. Future studies should aim to provide direct experimental evidence to move beyond plausible and indirectly supported hypotheses regarding the physiological functions of Ac-AAs. Reframing Ac-AAs from metabolic byproducts and biomarkers to central metabolic intermediates may enhance understanding of metabolic regulation and disease pathology.

## CRediT authorship contribution statement

**Dirk Weber:** Writing – original draft, Conceptualization. **Achim Bub:** Writing – review & editing, Conceptualization.

## Declaration of generative AI and AI-assisted technologies in the manuscript preparation process

The authors used ChatGPT (OpenAI) to assist with language refinement and improvement of text clarity. All content was carefully reviewed and verified by the authors, who take full responsibility for the accuracy and integrity of the manuscript. No other AI tools were used in the preparation of this work.

## Funding

This research received no external funding.

## Declaration of competing interest

All authors declare that the research was conducted in the absence of any commercial, financial, or other relationships that could be construed as a potential conflict of interest.

## Data Availability

No data was used for the research described in the article.
