# Supplementary material for: N-acetylated amino acids: An overlooked layer of metabolic regulation
Source: Mol Metab. 2026 Jul 16;111:102425. doi: 10.1016/j.molmet.2026.102425 (PMC13445479; doi:10.1016/j.molmet.2026.102425)
Supplement: Supplementary file 1 [file mmc1.docx]

**Supplementary Table 1. Metabolomics studies reporting N-acetylated amino acids in response to acute exercise and cold exposure in brown adipose tissue**

**N-acetylated amino acids: an overlooked layer of metabolic regulation**

Dirk Weber^1^, Achim Bub^1,2^

^1^ Institute of Sports and Sports Science, Karlsruhe Institute of Technology, Karlsruhe, Germany,

^2^ Department of Nutritional Epidemiology and Physiology, Max Rubner-Institute, Karlsruhe, Germany

**ORCID ID of the authors:**

| Dirk Weber | [0009-0004-0911-5485](https://orcid.org/0009-0004-0911-5485) |
| --- | --- |
| Achim Bub | [0000-0001-6907-5519](https://orcid.org/0000-0001-6907-5519) |

**Corresponding author:**

Dirk Weber

Karlsruhe Institute of Technology (KIT)

Engler-Bunte-Ring 15

76131 Karlsruhe (Germany)

[dirk.weber@kit.edu](mailto:dirk.weber@kit.edu)

**Keywords**: N-acetylated amino acids, Acetyl-CoA, Amino acids, Metabolic regulation

| **Study**  **(Author, Year)** | **Intervention** | **Species** | **Analytical**  **Platform** | **Sample**  **Timing** | **Altered**  **N-Acetylated Amino Acids** | **Proposed Interpretation** |
| --- | --- | --- | --- | --- | --- | --- |
| **Metabolomics studies reporting N-acetylated amino acids in response to acute exercise** | | | | | | |
| Nieman et al. (2013) | 3 × 2.5 h treadmill @ 70% VO₂max (3 d) | Humans (n = 15) | UHPLC–MS/MS (RP, ESI±, untargeted) + GC–MS (EI, untargeted) | Immediately after the last exercise session | **Serum**: Ac-Phe (+), Ac-Ala (+) | NA |
| Daskalaki et al. (2015) | 60 min treadmill and/or cycle ergometer (self‑paced) | Male humans (n = 3) | LC–MS (high-resolution Orbitrap, HILIC, ESI±, untargeted) | 1 h post exercise | **Urine**: Ac-Met (+), Ac-D-Trp (+), Indole‑3‑Ac‑Gln (+) | NA |
| Alzharani et al. (2020) | 2 × 2 h training sessions over 2 consecutive days (professional soccer players) | Male humans (n = 20) | LC–MS (high-resolution Orbitrap, HILIC, RP, ESI±, untargeted) | Immediately post exercise | **Plasma**: Ac-Glu (+), Ac-Arg (+), N2‑Ac‑L‑aminoadipate (+) | NA |
| Tabone et al. (2021) | Incremental treadmill test (1% grade; 10 km·h⁻¹ start, +0.3 km·h⁻¹/30 s to exhaustion) | Male humans (n = 40) | LC–HRMS (RP, HILIC, ESI±, untargeted) | Immediately post exercise | **Serum**: Ac-Phe (+), Ac-Tyr (+), Ac-Glu (+), Ac‑Trp (+), Ac‑Asp (-), Ac-Met (-) | Related to Acetyl-CoA |

| **Study**  **(Author, Year)** | **Intervention** | **Species** | **Analytical**  **Platform** | **Sample**  **Timing** | **Altered**  **N-Acetylated Amino Acids** | **Proposed Interpretation** |
| --- | --- | --- | --- | --- | --- | --- |
| Sato et al. (2022) | Treadmill running (60 min; 5% incline; 6 to 16 m·min⁻¹, +2 m·min⁻¹ every 2 min) | Mice | UPLC–MS/MS (high-resolution Orbitrap, untargeted) | Immediately post exercise | **Skeletal Muscle**: Ac-Gly (+), Ac-Phe (+), Ac‑Tyr (+), Ac‑Leu (+), Ac-Ile (+), Ac-Val (+), Ac‑Ser (+), Ac-Thr (+), Ac-Ala (+), Ac-Glu (+), Ac‑Tau (+), Ac‑Asp (-)  **Serum**: Ac-Gly (+), Ac-Thr (+), Ac-Ala (+), Ac‑Phe (+), Ac‑Tyr (+), Ac-Ile (+), Ac-Val (+), Ac‑Ser (+), Ac-Asn (+), Ac-Glu (+), Ac-Leu (+), Ac‑Arg (+), Ac‑Tau (+), Ac-Kyn (+), Ac-Cit (+), Ac‑Gln (-), Ac‑Pro (-)  **Liver**: Ac-Gly (+), Ac-Ile (+), Ac-Val (+), Ac-Glu (+), Ac-Arg (+), Ac-Tau (+), Ac-Cit (+), Ac-Met (-), Ac‑Gln (-), Ac-His (-)  **Heart**: Ac-Gly (+), Ac-Thr (+), Ac-Trp (+), Ac‑Leu (+), Ac-Ile (+), Ac-Val (+), Ac-Arg (+), Ac‑Cit (+), Ac-Met (-)  **Hypothalamus**: Ac-Gln (+), Ac-Phe (+), Ac-Leu (+)  **Epididymal White Adipose Tissue**: Ac-Gly (+), Ac-Phe (+), Ac-Leu (+), Ac-Arg (+), Ac-Asn (+), Ac‑Ser (+), Ac-His (+), Ac-Asp (-)  **Inguinal White Adipose Tissue**: Ac-Leu (+)  **Brown Adipose Tissue**: Ac-Tyr (+), Ac-Gly (+), Ac‑Thr (+), Ac-Leu (+), Ac-Ile (+), Ac-Val (+), Ac‑Arg (+), Ac-Cit (+), Ac-Ser (-), Ac-Glu (-) | NA |
| Fulghum et al. (2022) | Treadmill running to exhaustion | Female Mice | UPLC–MS/MS (high-resolution Orbitrap, RP, HILIC, ESI±, untargeted) | Immediately post exercise;  1 h post exercise | **Heart (immediately post exercise)**: Ac-Leu (+)  **Heart (1 hour post exercise)**: Ac-Thr (+) | NA |
| **Study**  **(Author, Year)** | **Intervention** | **Species** | **Analytical**  **Platform** | **Sample**  **Timing** | **Altered**  **N-Acetylated Amino Acids** | **Proposed Interpretation** |
| Weber et al. (2025) | 30 min bicycle ergometer at 50%/75% VO₂peak | Male humans (n = 17) | UPLC–MS/MS (high-resolution, RP, HILIC, ESI±, untargeted) | Immediately post exercise | **Plasma**: Ac-Met (+), Ac-Phe (+), Ac-Trp (+), Ac‑Leu (+), Ac-Val (+), Ac-Gln (+), Ac-Tyr (+), Ac‑Ile (+), Ac-Glu (+) | Acetyl-CoA buffer |
| Benedetti et al. (2025) | 24-h ultramarathon race | Male humans (n = 20) | UHPLC–MS (high-resolution Orbitrap, RP, ESI±, untargeted) | Immediately post exercise | **Serum**: Ac-Gly (+) | NA |
| Tang et al. (2025) | 30 min swimming @ 70–80% HRpeak or repeated 50-m sprints | Humans (n = 42) | LC–MS/MS (RP, ESI±, untargeted) | Immediately post exercise | **Serum**: Ac-Val (+) | Related to Acetyl-CoA |
| **Metabolomics studies reporting N-acetylated amino acids in response to BAT acclimation and deacclimation** | | | | | | |
| Mills et al. (2018) | BAT cold acclimation (4° C, 3 h) | Mice (n = 8) | UHPLC–HRMS (Orbitrap, HILIC, ESI±, untargeted) | Post acclimation | **BAT**: Ac-Asp (+), Ac-Met (+), Ac-Gln (+), Ac‑Leu (+), Ac-Ala (+), Ac-His (+), Ac-Arg (+), Ac‑Ornithine (+) | NA |
| Jung et al. (2021) | BAT cold acclimation (6 °C, 4 wk) | Mice (n = 17-20) | LC–HRMS (Orbitrap, HILIC, ESI±, untargeted) | Post acclimation | **BAT**: Ac-Cys (+), Ac-Met (+), Ac-Ser (+), Ac‑Asn (+), Ac-Asp (+), Ac-Glu (+), Ac-Gln (+), Ac‑Thr (+), Ac-Lys (+), Ac-Leu (+), Ac-Ala (+)  **Serum***: Ac-Met (+), Ac-Thr (+), Ac-His (+), Ac-Arg (+), Ac-Trp (+) | Acetyl-CoA buffer; sink for two-carbon units and CoA recycling; protein degradation |
| **Study**  **(Author, Year)** | **Intervention** | **Species** | **Analytical**  **Platform** | **Sample**  **Timing** | **Altered**  **N-Acetylated Amino Acids** | **Proposed Interpretation** |
| Verkerke et al. (2024) | BAT cold acclimation (6° C, 2 wk) | Mice (n = 5) | UHPLC–HRMS/MS (Orbitrap, HILIC, ESI±, untargeted) | Post acclimation | **BAT-derived extracellular fluids compared to WAT-derived extracellular fluids**: Ac-Glu (+), Ac‑Asp (+) | Involved in glutathione synthesis |
| Pileggi et al. (2026) | BAT cold deacclimation (4° C for 7 days) to 30° C | Mice (n = 10) | LC–MS/MS (triple quadrupole, RP, ESI±, targeted) | 48 h post deacclimation | **BAT**: Ac-Glu (−), Ac-Met (−), Ac-Ser (−), Ac‑Gln (−), Ac-Leu (−), Ac-Asp (−), Ac-Lys (+) | Protein degradation; acetyl-CoA buffer; regulation of histone acetylation; signaling molecules; individual functions |

The table summarizes reported alterations in Ac-AAs in response to high-intensity exercise and brown adipose tissue (BAT) acclimation and deacclimation. Studies investigating disease states or inborn errors of metabolism as well as studies focusing exclusively on N-acetylaspartate are not included. Standard three-letter abbreviations are used for amino acids. Plus signs (+) indicate increases, whereas minus signs (−) indicate decreases. The table is not intended to be comprehensive. *Calculated from Supplementary Data S1 as the difference in serum pool size between 6 °C and 22 °C using the reported statistical approach.
